# Evaluation of Oxford instability shoulder score, Western Ontario shoulder instability Index and Euroqol in patients with slap (superior labral anterior posterior) lesions or recurrent anterior dislocations of the shoulder

**DOI:** 10.1186/1756-0500-6-273

**Published:** 2013-07-15

**Authors:** Øystein Skare, Sigrud Liavaag, Olav Reikerås, Petter Mowinckel, Jens Ivar Brox

**Affiliations:** 1 Department of Orthopedic Surgery, Lovisenberg Diaconal Hospital, Lovisenberggaten 17, 0440, Oslo, Norway; 2 Department of Orthopedic Surgery, Sørlandet Hospital- Arendal, Sykehusveien 1, 4838, Arendal,Norway; 3 Department of Orthopedic Surgery, Oslo University Hospital-Rikshospitalet, Sognsvannsveien, 0027, Oslo, Norway; 4 Department of Pediatrics, Oslo University Hospital- Ullevål, 0407, Oslo, Norway

**Keywords:** Outcome measurements, Reliability, Agreement, Validity, Oxford instability shoulder score, Western ontario shoulder instability index, EuroQol, SLAP-lesions, Shoulder dislocations

## Abstract

**Background:**

Having an estimate of the measurement error of self-report questionnaires is important both for assessing follow-up results after treatment and when planning intervention studies. Specific questionnaires have been evaluated for patients with shoulder instability, but not in particular for patients with SLAP (superior labral anterior posterior) lesions or recurrent dislocations. The aim of this study was to evaluate the agreement, reliability, and validity of two commonly questionnaires developed for patients with shoulder instability and a generic questionnaire in patients with SLAP lesions or recurrent anterior shoulder dislocations.

**Methods:**

Seventy-one patients were included, 33 had recurrent anterior dislocations and 38 had a SLAP lesion. The patients filled in the questionnaires twice at the same time of the day (± 2 hours) with a one week interval between administrations. We tested the Oxford Instability Shoulder Score (OISS) (range 12 to 60), the Western Ontario Shoulder Instability Index (WOSI) (0 to 2100), and the EuroQol: EQ-5D (−0.5 to 1.0) and EQ-VAS (0 to 100). Hypotheses were defined to test validity.

**Results:**

ICC ranged from 0.89 (95% CI 0.83 to 0.93) to 0.92 (0.87 to 0.95) for OISS, WOSI, and EQ-VAS and was 0.66 (0.50 to 0.77) for EQ-5D. The limits of agreement for the scores were: -7.8 to 8.4 for OISS; -339.9 to 344.8 for WOSI; -0.4 to 0.4 for EQ-5D; and −17.2 and 16.2 for EQ-VAS. All questionnaires reflect the construct that was measured. The correlation between WOSI and OISS was 0.73 and ranged from 0.49 to 0.54 between the shoulder questionnaires and the generic questionnaires. The divergent validity was acceptable, convergent validity failed, and known group validity was acceptable only for OISS.

**Conclusion:**

Measurement errors and limitations in validity should be considered when change scores of OISS and WOSI are interpreted in patients with SLAP lesions or recurrent shoulder dislocations. EQ-5D is not recommended as a single outcome.

## Background

A number of self-report questionnaires have been developed to assess shoulder pain and disability from the patient’s perspective. The choice of a questionnaire may be based on factors such as study or diagnostic group, practical considerations regarding the ease of scoring, and the time to fill in the questionnaire as well as clinometric properties. A recent study reported that a general shoulder questionnaire was as good as the disease specific Western Ontario Rotator Cuff Index (WORC) and Oxford Shoulder Score (OSS) for rotator cuff disease [1]. Thus, the need for disease specific questionnaires for all different kinds of shoulder diagnoses can be questioned.

Shoulder instability can be defined as the loss of shoulder comfort and function due to undesirable translation of the humeral head on the glenoid [2]. From the patient perspective, shoulder instability may be defined as symptomatic abnormal motion of the glenohumeral joint which can present as pain or a sense of displacement (subluxation or dislocation) [3]. From a diagnostic point of view, instability is difficult to verify unless a dislocation has occurred. The latter is defined as a complete dissociation of the articular surfaces documented radiographically or by a manual reduction manoeuvre [4]. In patients with anterior shoulder dislocation, the main patho-anatomical finding is the Bankart lesions with avulsion of the labrum and the glenohumeral ligament from the anterior-inferior glenoid rim. A superior labral anterior posterior (SLAP) lesion of the shoulder is a relatively rare condition caused by injury or degeneration of the superior part of the glenoid labrum. Apprehension and loss of confidence are reported to be the major factors inhibiting sports activities and decreasing quality of life in patients with recurrent dislocations [5,6], while pain, popping, clicking, catching, weakness, stiffness, and instability (apprehension and loss of confidence) are reported in patients with SLAP lesions [7] Symptoms overlap in the two patients groups as those with recurrent dislocations also may experience pain, popping, clicking, stiffness, and weakness.

Several questionnaires have been designed to evaluate treatment of instability in the shoulder while specific questionnaires have not been published for patients with SLAP lesions. In the original study the Western Ontario Shoulder Instability Index (WOSI) was evaluated in 33 patients with shoulder instability, but not in particular for patients with recurrent shoulder dislocations [2]. Oxford Instability Shoulder Score (OISS) was evaluated in 53 patients diagnosed as having either unidirectional or multidirectional instability [6]. In a 5-year follow-up study of arthroscopic repair in patients with SLAP lesions [8], the clinical Rowe Score (1988 version) was used as the main effect variable. This score has been reported to have considerable limitations [9] and results [8] would have been strengthened applying a self-report outcome with acceptable measurement properties.

In absence of a disease-specific scoring system for SLAP lesions, existing questionnaires for shoulder instability [10], such as the OISS [11] and the WOSI [2], offer a possible alternative for the assessment of treatment effects in patients with SLAP lesions, because both conditions includes labral lesions that may cause similar symptoms.

The generic EuroQol provides an utility index for use in cost-effectiveness studies and for the comparison of results across different patient populations [12]. Most researchers advocate that studies of comparative effectiveness include a generic measurement of quality of life to allow for comparisons across patient populations [13,14].

The original studies of WOSI reported Interclass Correlation Coefficients (ICCs) for evaluation of reliability, but did not report agreement statistics [2]. Reliability describes the consistency of the test-retest variation within an individual relative to the variation between individuals in the group. The measurement error within a patient is best described by agreement parameters which estimate how close the results of repeated measures are. Agreement parameters have direct impact on reliability, effect size, responsiveness, and sample size calculations [15,16].

Self-report questionnaires are applied to evaluate the change in a patient or a group of patients following treatment or to evaluate the change between treatments in a clinical trial. It is important that both reliability and agreement are evaluated in methodological studies. Besides, other quality criteria of the instruments should be assessed. By example summarizing of the items in a scale is supported if the internal consistency or Chronbach’s alpha is high and indicate that the same concept is measured [17]. Correlation is often used to examine the association between different outcomes for evaluation of whether they can be used interchangeably. There is an ongoing debate about the interpretation of correlation as a measure of construct validity. The COSMIN (COnsensus-based Standards for the selection of health status Measurement INstruments) group have recommended to use hypotheses testing to assess various aspects of validity which include construct, convergent, divergent, and known group validity of an instrument [18].

The purpose of the present study was to cross-culturally adapt OISS and WOSI for use in Norwegian-speaking patients, and evaluate the agreement, inter-rater and intra-rater reliability, content- and construct validity of the Oxford Instability Shoulder Score, the Western Ontario Shoulder Instability Index, and the EuroQol in patients with recurrent anterior shoulder dislocations or SLAP lesions.

## Methods

### Study population and study design

Between November 2006 and August 2008, 103 patients referred for shoulder surgery at the Orthopaedic Department at Lovisenberg Diaconal Hospital in Oslo, Norway, were prospectively recruited. Eighty-five patients aged 16–60 years with a symptom duration of at least 3 months met the inclusion criteria for the study [9]. All patients signed an informed consent. The present study is approved by The Ethical Committee of Health Region South-East, Norway. Seventy-one patients (33 had recurrent anterior (at least two) dislocations and 38 had a SLAP lesion) were included. Patients with symptoms and signs suggesting a SLAP lesion were included if the lesion was confirmed on MRI arthrography [9]. Patients labelled SLAP lesion were not included if they had a history of shoulder dislocation. The exclusion criteria for the study were posterior or multidirectional dislocations; inability to complete the questionnaires; previous surgery for SLAP injuries or instability in the same shoulder; rheumatic disease affecting the symptomatic shoulder; pain referred from the cervical or thoracic spine; and severe somatic or psychiatric disorders. All included patients gave a written informed consent.

The patients completed OISS, WOSI, the 1988 version of Rowe Score, and EuroQol questionnaire twice, at the same time of the day with a one week interval between administrations. The test-retest period was chosen to reduce recall bias. One patient was excluded at retesting because he reported major changes in his activity level, and deterioration between tests.

### Questionnaires

OISS is a disease-specific health-related quality-of-life self-report questionnaire, for use in patients with shoulder instability [6]. Several names and abbreviations have been used synonymously, such as Oxford Instability Score (OIS) [19] and Shoulder Instability Questionnaire (SIQ) [20]. The instrument consists of 12 questions, each of which had five response alternatives, ranked from least to most difficult (1–5 points). The items cover episodes of instability, daily activities, pain, work, social life, sports/hobbies, attention to the shoulder problem, lifting, and lying positions with a total possible score ranging from 12 (best function) to 60 (worst function) [6].

WOSI consists of 21 self-report questions representing four domains (sports, recreation/work, lifestyle and emotions). Each question is answered on visual analogue scale ranging from 0 (best) to 100 (worst). The total score ranges from 0 (best) to 2100 (worst) [2].

The EuroQuol is a generic health-related quality-of-life instrument [12,21,22]. EQ-5D consists of five domains (mobility, self-care, usual activities, pain/discomfort, and anxiety/depression), with three levels corresponding to no problem, some problem and an extreme problem. The responses are transformed into a utility index and are then classified into 243 (3^5^) health states ranging from the best imaginable state (1.0), and worst possible score (0.59). EQ-VAS estimates generic health status by using a visual analogue scale from 0 (worst possible) to 100 (best possible).

For assessment of the correlation between scores we also included the 1988 version of the clinical Rowe Score [9,23].

### Translation

The EQ-5D was already cross-culturally adapted for use in Norwegian-speaking population [24]. Cross-cultural adaptations of the Norwegian versions of OISS and WOSI was conducted according to the procedures described in the literature [25,26]. Forward translation of OISS and WOSI was done by two bilingual medical doctors, one bilingual nurse and one bilingual medical doctor and professional translator. Two had Norwegian as their native language and two had English as their native language. The translations were done independent of each other and then compared. The Norwegian versions were then back-translated into English by a professional translator. The back-translated versions were then reviewed and inconsistencies of the items of OISS and WOSI were discussed and approved in a consensus meeting with the four translators.

### Statistical analysis

The study was planned to have a sample size of at least 50 patients, which is the general recommendation given by Altman for a methods comparison study [27]. All patients had chronic complaints and we assumed that diagnostic group did not influence agreement statistics. For reliability and validity evaluation we could not exclude that diagnostic group may influence results and some exploratory analyses were performed in each diagnostic group.

Age, duration of symptoms, and number of dislocations were described by median (range) while numbers (percentages) are reported for gender, manual labour, physical activity level, and whether the dominant shoulder was involved. Means (SD) were used for descriptive statistics for total scores and domain scores of WOSI and for the total scores of OISS, EQ-5D and EQ-VAS.

The data of the descriptive statistics data followed a normal distribution. Differences between groups were compared by Student’s two-sample t-test, Chi-square was used for categorical variables. Minimum and maximum scores for individual items, domain and total scores were examined for possible floor and ceiling effects, which were considered to be present if more than 15% of respondents achieved the highest or lowest score, respectively.

*Internal consistency* describes the correlations among items measuring the same concept on questionnaire (sub)scales [17], A Chronbach’s alpha between 0.70 and 0.95, indicates strong correlation between items in a scale [17,18]. We calculated the internal consistency for the total scores and domain scores.

*Test* –*retest reliability* is commonly tested by ICC. which combines the within and between patient variation from 0 (no reliability) to 1 (perfect reliability). According to Terwee et al., an ICC > 0.70 is considered to be acceptable [17]. We used a two-way random single measure (ICC 2.1), with a 95% confidence interval for the total score and for the domains [17,28].

*Agreement* describes the within patient measurement error, and indicates how close the scores of repeated measurements are to one another [17]. Statistical methods to estimate measurement error include standard error of measurement (SEM), limits of agreement (LoA), and minimal detect able change (MDC) which equals the repeatability coefficient [17,18,29]. SEM is recommended as the measure of agreement [18]. It can be estimated as SEM_consistency_ (SD√(1-ICC)) or SEM_agreement_ (within- subject standard deviation (S_w_)). The latter is obtained by extracting the square root of the residual mean square, using one-way ANOVA with subjects as the factor [30,31]. While the SEM_consistency_ include both between and within-subject variations, SEM_agreement_ takes only the within-subjects variation into account. The COSMIN checklist for does not give information about a particular version of SEM [18,32,33]. In the present study, we estimated SEM_agreement_, minimal detectable change (SEM × 1.96√2) and limits of agreement (mean individual difference ± SD of differences) with 95% confidence interval. We constructed agreement plots according to Bland and Altmann [34].

*Validity* describes whether an instrument measures what it is intended to [13].

*Content validity* indicates that the concepts of interest are comprehensively represented by the items in the questionnaire [32,35]. Terwee et al. recommended that authors should provide clear descriptions aims of the questionnaire, the target population, the concepts intended to be measured, item selection, reduction and interpretability [17]. According to the COSMIN checklist [32], content validity should be assessed by making a judgment about the *relevance* and *comprehensiveness* of the items. Patients or experts should be asked whether they missed any items. In the present study, this was checked during the cross cultural adaptation process and by assessing floor and ceiling effects of the domains and single questions of the instruments [17,32]. Large floor and ceiling effects suggest that content validity is low. Floor and ceiling effects were considered apparent if 15% or more of the responders had the lowest or the highest possible score, respectively.

*Construct validity* means that questionnaire measures the relevant constructs [33]. The COSMIN checklist recommends to use hypotheses to test relationships with other instruments or differences among relevant groups [32]. Construct validity is considered acceptable when at least 75% of the hypotheses are accepted [17]. To admit comparison of construct validity with other studies not using hypotheses, Pearsons correlation coefficient between OISS, WOSI, EQ-5D, EQ-VAS and the 1988 version of Rowe Score was obtained.

There are several aspects of construct validity which include *convergent*, *divergent*/*discriminant*, and *known group* validity. *Convergent validity* reflects correlation with other instruments that measure the same properties [39]^12^. Convergent validity for hypotheses 1 to 8 was tested using Pearsons correlation coefficient. R > 0.70 was regarded as positive correlation [17]. *Divergent validity*/*discriminant validity* evaluates whether concepts of measures that are supposed to be unrelated are in fact unrelated [36]. Tests can be invalidated by too high correlations with other tests they were intended to differ [36]. In the present study the formula r_xy_ /√(r_xx_ * r_yy)_ was used to test discriminant validity [36]. Hypotheses 12 and 13 were tested using the formula r_xy_ /√(r_xx_ * r_yy)_,where r_xy_ is the correlation between EQ-5D and OISS and WOSI, r_xx_ is the ICC of OISS or WOSI, and the r_yy_ is the ICC of EQ-5D. A result <0.85 is considered to indicate acceptable discriminant validity [36]. *Known group validity* describes the relationships among different groups (age, gender, diagnosis, etc.). Independent sample *t*- tests were used to test known group validity for hypotheses 9 to 11.

#### Hypotheses

*Convergent validity* (*positively correlated means r* >*0*.*70*)

1. WOSI should be positively correlated OISS.

2. WOSI should be positively correlated with Rowe Score.

3. OISS should be positively correlated with Rowe Score.

4. WOSI part B (Sports/Recreation/Work) should be positively correlated with question 8 of OISS: “During the last four weeks, how much has the problem with your shoulder interfered with your sporting activities or hobbies?”

5. WOSI part D (Emotions) should be positively correlated with question 9 of OISS: “During the last four weeks, how often has your shoulder been «on your mind”- how often have you thought about it?”

6. WOSI part C (Lifestyle) should be positively correlated with question 12 of OISS: “During the last four weeks, have you avoided lying in certain positions in the bed at night because of your shoulder?”

7. WOSI part A (Physical symptoms) should be positively correlated with question 3 of OISS: “During the last three months, how would you describe the worst pain you have had from your shoulder?”

8. Question 1 of OISS — “During the last six months, how many times has your shoulder slipped out of joint (or dislocated)?” — should be correlated with question 8 of WOSI part A: “How much feeling of instability or looseness do you experience in your shoulder?”

Known group validity

9. OISS should be the same for patients < 45 and > 45 years old.

10. WOSI should be the same for patients< 45 and > 45 years old.

11. The scores of the SLAP group should be negatively correlated (R < 0.70) with the scores of the instability group of question 1 of OISS: “During the last six months, how many times has your shoulder slipped out of joint (or dislocated)?”.

*Divergent*/*discriminant validity*

12. The discriminate validity between OISS and EQ-5D should be < 0.85.

13. The discriminate validity between WOSI and EQ-5D should be < 0.85. The analysis was performed using Statistical Analysis System software (SAS, version 9.2, SAS Institute Inc., Cary NC, USA).

## Results

### Demographics

Fifty men (70.4%) and 21 women (29.6%) were included for further analysis in this study (Table 1). There were no differences in baseline characteristics among the 14 patients who were excluded, compared with those patients who were included. The patients in the instability group were younger than the SLAP group and had a median of 10 (range 2 to 40) dislocations. The two diagnostic groups did not differ on the mean scores of the questionnaires.

**Table 1:** Descriptive statistics

	**SLAP**	**Instability**
Males/females [n]	28/10	22/11
Age (median [range])	40 (16–60)	25 (19–54)
Duration of symptoms median months (range)	23 (4–132)	36 (10 – 360)
Manual labour n (%)	21 (55.3)	14 (42.4)
Physical activity		
competition	4 (10.5)	5 (15.2)
weekly or more	20 (52.6)	20 (60.6)
none	14 (36.8)	8 (24.2)
Shoulder involved; right/left	27/11	14/19
Dominant shoulder involved n (%)	26 (68)	15 (45)
Number of dislocations median (range)	0	10 (2 – 40)
WOSI total score	1081.7 (382.8)	1025.8 (438.9)
OISS total score	37.4 (7.6)	33.7 (10.4)
EQ-5D index	0.65 (0.22)	0.76 (0.25)
EQ-VAS	71.2 (15.0)	72.7 (21.3)
Rowe total score	66.9 (10.6)	63.9 (11.0)

*SLAP* superior glenoid labrum lesions, *WOSI* Western Ontario shoulder Instability Index, *OISS* Oxford Instability Shoulder Score, *EQ*-*5D*, *EQ*-*VAS*, EuroQol. Rowe score; 1988 version. Scores are given for first evaluation.

### Cross cultural validity

The EuroQol instrument was already cross-culturally adapted into Norwegian [24]. The relevance and translations of items of OISS and WOSI were discussed and approved by the consensus group. The translated versions of OISS and WOSI adequately reflected items in the original-language versions.

### Internal consistency

Chronbach’s alpha for the total scores of OISS, WOSI, and EQ-VAS was ranged from 0.94 to 0.96 (Table 2). There Chronbach’s alpha was 0.79 for EQ index and ranged from 0.87 to 0.96 for the domains of WOSI (Table 2).

**Table 2:** Agreement and reliability statistics - total scores

	**1.test Mean (SD)**	**2.test Mean (SD)**	**Mean difference (95% CI)**	**Limits of agreement (LoA)**	**Minimal detectable change (95% CI)**	**ICC (2.1) (95% CI) **^**†**^	**Standard error of measurement (SEM**_**agreement**_**)**	**Chronbach’s alpha**
OISS (12to 60)	35.7 (9.1)	35.4 (8.9)	0.3 (−0.7 to 1.2)	(−7.8 to 8.4)	8.1 (5.4 to 10.8)	0.90 (0.84 to 0.94)	2.9	0.95
WOSI (0 to 2100)	1055.7 (407.8)	1050.3 (444.6)	5.4 (−35.6 to 36.4)	(−333.9 to 344.8)	339.3 (227.0 to 451.8)	0.92 (0.87 to 0.95)	122.4	0.96
EQ-5D (−0.53 to 1)	0.70 (.24)	0.71 (0.24)	−0.01 (−0.06 to 0.04)	(−0.4 to 0.4)	0.4 (0.3 to 0.5)	0.66 (0.50 to 0.77)	0.1	0.79
EQ-VAS (0 to 100)	71.9 (18.1)	72.3 (18.7)	−0.42 (−2.4 to 1.6)	(−17.1 to 16.2)	16.6 (11.2 to 22.2)	0.89 (0.83 to 0.93)	6.0	0.94

*SD* standard deviation, *ICC* (*2*.*1*) interclass correlation version 2.1 for measuring correlation between test and retest. Agreement estimated by the difference between test and retest, the limits of agreement (LoA), the standard error of measurement (SEM_agreement_), and minimal detectable change (*MDC*) with 95% confidence interval. Chronbach’s alpha (internal consistency) are given for the 2.test. 95% CI (confidence interval) for paired t-test under null hypothesis = no difference between test and retest score.

† P< .0001 for all ICC (interclass correlation coefficient version 2.1).

### Test-retest reliability

ICC ranged from 0.89 (95% CI 0.83 to 0.93) to 0.92 (0.87 to 0.95) for the total scores of OISS, WOSI, and EQ-VAS and was 0.66 (0.50 to 0.77) for EQ-5D (Table 2). For the domains of WOSI, ICCs ranged from 0.77 (0.65 to 0.85) to 0.92 (0.88 to 0.95) (Table 3). ICC ranged from 0.01 (−0.22 to 0.24) to 0.75 (0.63 to 0.84) for the domains; walking, personal care, and daily activities of EQ-5D (Table 4).

**Table 3:** Agreement statistics, internal consistency and content validity for the domains of the WOSI 1. and 2.test

**Outcome (scores)**	**Median (min., max.)**	**Limits of agreement (LoA)**	**ICC (2.1)**^**† **^**(95% CI)**	**Floor effects %**	**Ceiling effects %**	**Minimal detectable change (MDC) (95% CI)**	**Standard error of measurement (SEM**_**agreement**_**)**	**Chronbach’s alpha**
Physical symptoms	452	(−171.4 to 54.3)	0.92 (0.88 to 0.95)	0	0	162.9 (108.9 to 216.9)	58.8	0.96
	(20, 897)							
Sports, recreation and work	243 (21, 398)	(−96.6 to 118.5)	0.82 (0.72 to 0.88)	0	0	107.6 (72.0 to 143.2)	38.8	0.90
Lifestyle	190 (4, 399)	(−103.5 to 106.5)	0.87 (0.81 to 0.92)	0	0	105.0 (70.2 to 139.8)	37.8	0.93
Emotions	206 (2, 299)	(−91.2 to 116.2)	0.77 (0.65 to 0.85)	0	0	103.7 (69.3 to 138.1)	37.4	0.87

*ICC* (*2*.*1*), interclass correlation version 2.1 for measuring correlation between test and retest. Agreement estimated by the difference between test and retest, minimal detectable change (*MDC*) with 95% confidence interval, standard error of measurement (*SEM*_*agreement*_), and limits of agreement (LoA). Chronbachs alpha (internal consistency) are given for the 2.test. Content validity is measured by floor and ceiling effects.

95% CI (confidence interval) for paired t-test under null hypothesis = no difference between test and retest score.

^†^*P*< .*0001 for all ICC* (*interclass correlation coefficient version 2*.*1*).

**Table 4:** Agreement statistics and content validity for the domains of the EQ-5D 1.and 2.test

**Outcome (scores)**	**Median (min. max.)**	**Limits of agreement (LoA)**	**Floor effects %**	**Ceiling effects %**	**Minimal detectable change (MDC) (95% CI)**	**ICC**^**† **^**(95% CI)**	**Standard error of measurement (SEM**_**agreemen**_**)**
Walking	0.00 (0, 1)	(−0.30 to 0.35)	97.2	0.0	0.3 (0.2 to 0.4)	0.01 (−0.22 to 0.24)	0.2
Personal care	0.00 (0, 1)	(−0.63 to 0.61)	84.5	0.0	0.6 (0.4 to 0.8)	0.65 (0.49 to 0.77)	0.2
Daily activities	1.00 (0, 2)	(−0.85 to 1.04)	29.6	5.6	0.9 (0.6 to 1.2)	0.63 (0.47 to 0.75)	0.3
Pain/discomfort	1.00 (0, 2)	(− 0.73 to 0.81)	22.5	9.9	0.8 (0.5 to 1.1)	0.73 (0.60 to 0.82)	0.3
Anxiety/depression	0.00 (0, 1)	(−0.66 to 0.57)	79.1	0.0	0.6 (0.4 to 0.8)	0.75 (0.63 to 0.84)	0.2

*ICC* (*2*.*1*), interclass correlation version 2.1 for measuring correlation between test and retest. Agreement estimated by the difference between test and retest, minimal detectable change (*MDC*) with 95% confidence interval, limits of agreement (LoA), and standard error of measurement (SEM_agreemen_). Content validity is measured by floor and ceiling effects.

95% CI (confidence interval) for paired t-test under null hypothesis = no difference between test and retest score.

^†^*p* < 0,0001 for all ICC’s (interclass correlation coefficient version 2.1) except for the domain walking (*p* = 0.93).

### Agreement

**Figure 1:**
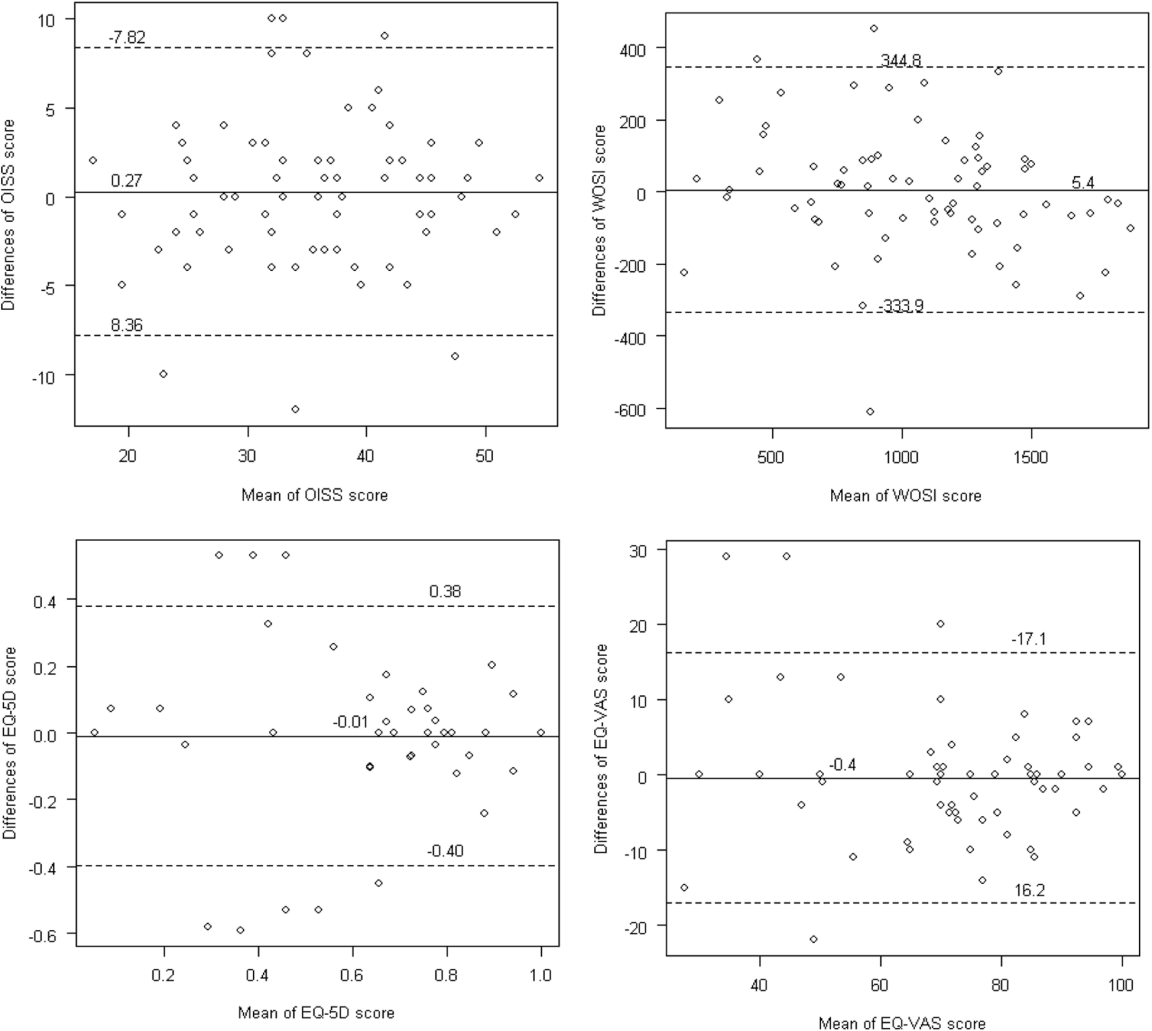
**Limits of agreement plots.** Average of 1. and 2.test total scores of OISS, WOSI EQ-5D, and EQ-VAS. On each plot, the central line represents the mean of the scores and the flanking lines represents the 95% limits of agreement.

There were no significant differences between the first and second administration of the scores (Table 2) or between diagnostic groups (Table 1). SEM_agreement_ for the total score were 2.9 for the OISS; 122.4 for the WOSI; 0.1 for the EQ-5D; and 6.0 for the EQ-VAS (Table 2). The minimal detectable change for the total scores was 8.1 points for the OISS; 339.3 points for the WOSI; 0.4 points for the EQ-5D; and 16.6 points for the EQ-VAS (Table 2). For the total scores, the limits of agreement were −7.8 to 8.4 for the OISS; -333.9 to 344.8 for the WOSI; -0.4 to 0.4 for the EQ-5D; and −17.1 to 16.2 for the EQ-VAS (Table 2). For the domains of the WOSI and the EQ-5D, the results are given in Tables 3 and 4 respectively. The limits of agreement plots are shown in Figure 1.

### Content validity

The OISS, the WOSI, and the EuroQol reflected the construct to be measured. However, in this study, 4 of the 38 patients with SLAP lesions reported experiencing shoulder dislocation over the previous 6 months (Question 1, OISS). There were no floor and ceiling effects for the total score of OISS or the single item scores, the domain scores, and the total score of WOSI. For single items of OISS, floor effects were observed for question 1 (shoulder instability) in the SLAP group and in both groups for question 2, 7, and 12, and ceiling effects for question 7, 9, 10, and 12. For EQ-5D the floor effects ranged from 22% to 97% (Table 4).

### Construct validity

There were no missing items. The correlation between WOSI and OISS was; 0.64 (95% CI 0.41 to 0.80)for the SLAP group and 0.80 (95%CI 0.62 to 0.69) for recurrent dislocations. The correlations between the specific questionnaires and EQ-5D and EQ-VAS ranged from −0.27 (95% CI −0.54 to 0.05) to −0.59 (95% CI – 0.79 to 0.32) with r < 0.60 for both diagnostic groups. The Rowe score correlated −0.42 (95% CI −0.67 to −0.09) with WOSI for the SLAP group, and −0.59 (95% CI −0.76 to −0.33) for the recurrent dislocation group, r < 0.60 in both groups. The correlation between the Rowe score and OISS was −0.30 (95% CI −0.58 to 0.05) for the recurrent dislocation group, and −0.45 (95%CI −0.67 to −0.15) for the SLAP group r < 0.60 in both groups.

### Convergent validity

Hypotheses (1 to 8) failed (r > 0.70 only for hypothesis 1).

### Known group validity

Hypotheses (9 to 11) failed (p<0.05 only for OISS, hypothesis 9).

### Divergent/discriminant validity

Hypotheses 12 and 13 were accepted, with r = 0.58 and 0.57, respectively.

## Discussion

This study contributes to the knowledge about the reliability, agreement and validity of OISS, WOSI, EQ-5D, and EQ-VAS in patients with SLAP-lesions or recurrent anterior shoulder instability.

### Internal consistency

The internal consistency for OISS was slightly different from that reported by the developers [6]. Because there are no domains in OISS, the internal consistency covers the total score of all 12 items. For WOSI findings are in keeping previous versions [37,38], but higher than those reported for the domain lifestyle [39,40]. For EQ-VAS, the Chronbach’s alpha was in keeping with the results of Adobor et al. [41], slightly lower for EQ-5D.

### Reliability

ICC for the OISS was comparable with the results of Moser et al. [42]. For the WOSI, it was in accordance with the original version and later published versions [2,37,38], and for EQ-VAS it was slightly higher than that of the original version [12]. In contrast to previous studies the reliability of EQ-5D was not acceptable in the present study [12,41].

### Agreement

Agreement of OISS, reported by standard error of measurements and minimal detectable change were in the same range as reported by Moser et al. [42]. When interpreting minimal detectable change in a patient, a difference of test and retest score of < 8.1 is within measurement error. The same interpretation can be made for the other instruments reading Tables 2, 3 and 4. The measurement error found for WOSI (Tables 2 and 3) is larger than the findings of Cacchio et al. [37], reporting SEM_consistency_ of 71 points and minimal detectable change of 196 points. The differences may be attributed the use of different versions of SEM and methods to calculate minimal detectable change.

Because ICC depends on both within- and between-subjects variation, it can be misleadingly high, and SEM correspondingly lower, if the between-subjects variation is high. As pointed out by Weir [28], also different versions of the ICC can result in different estimates and substantially affect the size of the SEM. This inconsistency represents a problem for comparison between studies. SEM estimated as the square root of the mean square error term from the ANOVA avoids this problem, although the results will differ depending on the application of a one-way model or a two-way model as well as specification of fixed effects or random (individual) effects. The limits of agreement is not affected by the various methods used for calculating the ICC and SEM, and represents a uniform estimate of the measurement error that is easier to compare between studies. As shown in Tables 2 and 3, the limits of agreement were considerable for all questionnaires. For EQ-5D limits between −0.4 and 0.4 on a scale ranging from - 0.53 to 1 means that this index is imprecise for estimating true change in an individual patient.

### Content validity

In agreement with previous studies [2,6,12,37-43], all the questionnaires reflected the constructs to be measured. One of the aims of the present study was to evaluate the questionnaires for use in studies with patients with SLAP lesions, as the original versions of the OISS and WOSI were developed for use in patients with instability. Question 1 in OISS — *During the last six months*, *how many times has your shoulder slipped out of joint* (*or dislocated*)? — is not expected to be relevant for patients with superior labral tears (SLAP II lesions). However, 4 of 38 patients answered that their shoulder had slipped out of the joint, suggesting that they had the experience that this had occurred, or that they did not understand the question. Unfortunately, we did not interview the patients about how they interpreted this question.

The good content validity of the total scores of OISS and WOSI was supported by the absence of floor and ceiling effects for these questionnaires. Although single items of OISS had considerable floor and/or ceiling effects for both diagnostic groups, there were no floor or ceiling effects for single items of WOSI using the 15% definition. As noted by Ekeberg et al., agreement parameters can be overestimated when floor and ceiling effects appear, as an extreme value is more likely to be repeated in a retest [1]. The considerable floor effects of EQ-5D call into question the use of this generic self-report index in the population examined. The floor effects of EQ-5D suggest that health-related quality is not much affected by a SLAP-lesion or recurrent dislocation and that a specific questionnaire should be preferred. The use of EQ-5D cannot be recommended for use in cost-effectiveness studies in the present patient population. It may be better suited for shoulder patients who are expected to be more disabled, by example patients with comminute fractures of the humeral head [44].

### Construct validity

In the present study, the construct validity was evaluated using both the correlation between instruments and the new criteria of the COSMIN group [32]. Previous studies have using correlation have reported good construct validity for OISS [6,20], WOSI [2,20,37-40], and EuroQol [21,22,24,41,45]. We found WOSI and OISS to be acceptably correlated for both diagnostic groups, which suggests that the self-report questionnaires can be used interchangeably. The EQ-5D, EQ-VAS, and Rowe score correlated < 0.60 with the specific questionnaires in both groups, which suggests that different constructs are measured. Applying the COSMIN checklist, OISS was acceptable for the two aspects of construct validity, but none of the questionnaires had acceptable convergent validity, but the use of hypotheses for the evaluation of construct validity is preferable, according to the COSMIN group [32] and to Guyatt [35]. The use of specific hypotheses also reduces the risk of bias, as stated by Terwee et al. [17], by avoiding the possibility of the retrospective construction of alternative explanations for the observed correlations. Nevertheless, the number of hypotheses applied can influence conclusions about validity.

### Advantages and limitations of the study

The main advantages of the present study, in comparison with previous studies, are the evaluation of the scores according to recommendations in the COSMIN checklist. Although patients with SLAP lesions and patients with instability are comparable on most items, differences appeared [9]. One limitation of the current study is that the sample size of each diagnostic group is small; however no major differences appeared between groups. For future studies, including responsiveness, larger studies for each diagnostic group are recommended.

## Conclusion

The measurement error and aspects of construct validity should be considered when OISS and WOSI are used in patients with recurrent shoulder dislocation and patients with SLAP-lesions. EQ-5D is not to be recommended as a single outcome instrument. The different methods for estimating SEM is a challenge when comparing measurement errors across studies.

## Competing interests

The authors declare that they have no competing interests.

## Authors’ contributions

ØS participated in the design of the study, drafted the manuscript, and evaluated patients for inclusion and follow-up exams. SL participated in the design of the study, presided the translation- and cross cultural adaptation process and helped to draft the manuscript. OR participated in the design of the study, contributed in monitoring the trial and drafting the manuscript. PM participated in the design of the study, planned and preformed the statistical analysis. JIB participated in the design of the study, monitored the trial, contributed to the translation- and cross cultural adaptation process, and helped with drafting the manuscript. All authors read and approved the final manuscript.
